# Dissociating Cognitive Processes During Ambiguous Information Processing in Perceptual Decision-Making

**DOI:** 10.3389/fnbeh.2020.00095

**Published:** 2020-07-13

**Authors:** Vladimir A. Maksimenko, Alexander Kuc, Nikita S. Frolov, Marina V. Khramova, Alexander N. Pisarchik, Alexander E. Hramov

**Affiliations:** ^1^Institute of Information Technologies, Mathematics and Mechanics, Lobachevsky State University of Nizhny Novgorod, Nizhny Novgorod, Russia; ^2^Center for Technologies in Robotics and Mechatronics Component, Innopolis University, Innopolis, Russia; ^3^Faculty of Information Technologies, Saratov State University, Saratov, Russia; ^4^Center for Biomedical Technology, Technical University of Madrid, Madrid, Spain

**Keywords:** perceptual decision-making, ambiguous stimuli, sensory processing, decision-making, top-down control, disambiguation process

## Abstract

Decision-making requires the accumulation of sensory evidence. However, in everyday life, sensory information is often ambiguous and contains decision-irrelevant features. This means that the brain must disambiguate sensory input and extract decision-relevant features. Sensory information processing and decision-making represent two subsequent stages of the perceptual decision-making process. While sensory processing relies on occipito-parietal neuronal activity during the earlier time window, decision-making lasts for a prolonged time, involving parietal and frontal areas. Although perceptual decision-making is being actively studied, its neuronal mechanisms under ambiguous sensory evidence lack detailed consideration. Here, we analyzed the brain activity of subjects accomplishing a perceptual decision-making task involving the classification of ambiguous stimuli. We demonstrated that ambiguity induced high frontal θ-band power for 0.15 s post-stimulus onset, indicating increased reliance on top-down processes, such as expectations and memory. Ambiguous processing also caused high occipito-parietal β-band power for 0.2 s and high fronto-parietal β-power for 0.35–0.42 s post-stimulus onset. We supposed that the former component reflected the disambiguation process while the latter reflected the decision-making phase. Our findings complemented existing knowledge about ambiguous perception by providing additional information regarding the temporal discrepancy between the different cognitive processes during perceptual decision-making.

## 1. Introduction

Perceptual decision-making represents choosing a course of action based on available sensory evidence (Heekeren et al., 2008). This process implies the evaluation of sensory information to make a decision and translate it into behavior. Since our sensory system tends to be noisy and stimuli are often represented ambiguously, the final decision substantially depends on the interpretation of sensory information (Heekeren et al., 2004).

Earlier studies on perceptual decision-making in rodents and monkeys used implanted micro-electrodes and identified spatially localized neuronal activity correlated with their behavioral performance. At the same time, the limited number of recording sites used precluded the detection of interaction between distinct brain regions coordinating perceptual decisions (Hanks and Summerfield, 2017). More recent work reported on recordings from multiple units in the sensory, parietal, prefrontal, and motor cortices during a perceptual decision-making task (Siegel et al., 2015). The authors demonstrated that perceptual decisions resulted from complex temporal dynamics, including coupling between the frontal and posterior cortices. Large-scale cortical interactions play a critical role in human perceptual decision-making. After reviewing a large number of neuroimaging studies, Siegel et al. (2011) concluded that perceptual decisions in humans relied on neuronal activity in the high-frequency γ (>50 Hz) and low-frequency β (15–30 Hz) bands. They specified that the localized γ-band activity in the sensorimotor cortex reflected information encoding and motor planning, while the large-scale β-band activity across widespread cortical areas coordinated the activity of these local networks.

According to the review (Siegel et al., 2011), perceptual decision-making includes two stages, sensory information processing and decision-making. Mostert et al. (2015) further demonstrated that these stages involved different brain areas in different time intervals. While the sensory processing takes place in the occipital cortex during 130–320 ms post-stimulus onset, the decision-related process is longer and activates parietal and frontal areas. The other studies (Philiastides and Sajda, 2005; Wyart et al., 2012; Kelly and O'Connell, 2013) reported on temporal dissociation between the sensory processing and decision-making stages for different types of stimuli. Philiastides and Sajda (2005) analyzed the influence of sensory evidence quality on the neuronal activity during the processing stage. The authors concluded that the evidence accumulation process began after early visual perception and lasted 290–440 ms depending on the strength of the evidence. In our recent study (Maksimenko et al., 2019), we considered the decision-making stage of the ambiguous stimuli classification task and observed that the emergence of a large-scale frontoparietal network in the β-band preceded the perceptual decisions. We reported that neither the network structure nor the duration of its formation depended on the stimulus ambiguity. Thus, we supposed that the formation of a large-scale β-band network served for the integration of decision-relevant sensory information into the decisions and their further translation into the behavioral response. The extraction of decision-relevant features, in turn, relied on the earlier processing stages, and this process depended on the quality and strength of the sensory evidence.

To better understand the evidence accumulation process, we consider a perceptual decision-making task implying the classification of ambiguous (bistable) visual stimuli with different degrees of ambiguity. When the stimulus ambiguity is low, its morphology reflects one of two possible interpretations. On the contrary, when the stimulus ambiguity is high, the observer experiences difficulty in making a decision and hence takes more time to accumulate the evidence. Following Philiastides and Sajda (2005) and Maksimenko et al. (2019), we analyzed the event-related spectral perturbations (ERSP) during the sensory processing (0.5 s post-stimulus onset) and response formation (0.3 s before response) stages. We demonstrated that increasing ambiguity resulted in a higher frontal θ-band power for 0.15 s post-stimulus onset. This could indicate an increased reliance on top-down processes while processing ambiguous stimuli compared to when processing unambiguous stimuli. These top-down processes might be specifically related to expectations, memory, and conflict resolution. Ambiguous processing also caused high occipito-parietal β-band power for 0.2 s and high fronto-parietal β-power for 0.35–0.42 s post-stimulus onset. We supposed that the former component reflected the disambiguation process while the latter reflected the decision-making. Our findings complemented existing knowledge about ambiguous stimulus processing by providing additional information regarding the temporal discrepancy between the different cognitive processes during perceptual decision-making.

## 2. Methods

### 2.1. Participants

Twenty healthy subjects (eleven males and nine females) aged from 21 to 36 (M = 26.1, SD = 4.6) with normal or corrected-to-normal visual acuity participated in the experiments on a voluntary basis. All of them provided written informed consent in advance. Participants were familiar with the experimental task and had not participated in similar experiments in the previous 6 months. The experiments were performed under the Declaration of Helsinki and approved by the local Research Ethics Committee of Innopolis University.

### 2.2. Visual Stimuli

**Figure 1:**
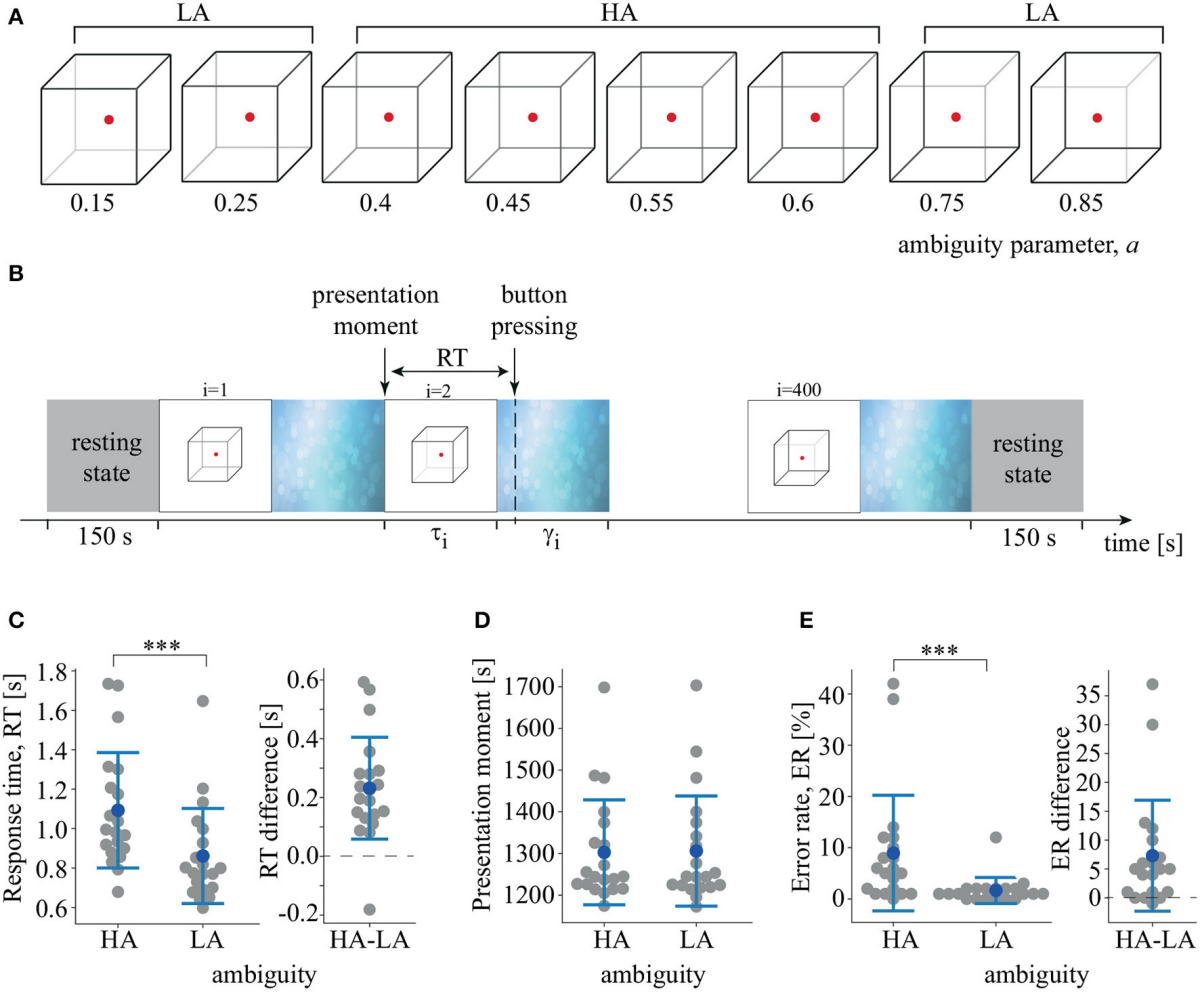
**(A)** Set of visual stimuli, Necker cubes, with different degrees of ambiguity, *a*. **(B)** Schematic illustration of the experimental sessions. τ_*i*_ is the duration of *i*-th stimulus presentation, γ_*i*_ is the time of the abstract image presentation between *i*-th and (*i* + 1)-th stimuli. RT is the response time. **(C)** RT to LA and HA stimuli and distribution of the pairwise differences (^***^p < 0.001, *t*-test). **(D)** Median presentation times of LA and HA stimuli. **(E)** Error-rate (ER) of LA and HA stimulus processing and distribution of the pairwise differences (^***^p < 0.001, Wilcoxon test). Group data are shown as means±SD and individual values.

We used a Necker cube as an ambiguous visual stimulus (Hramov et al., 2017; Kornmeier et al., 2017). A subject without any perceptual abnormalities interprets a Necker cube as a left- or right-oriented 3D-object, depending on the contrast of the inner edges. The contrast of three middle lines centered in the left middle corner was used as a control parameter *a*, where *a* = 1 and *a* = 0 corresponded to 0 (black) and 255 (white) pixel luminance according to 8-bit grayscale palette. Therefore, we defined the control parameter as *a* = *g*/255, where *g* was the brightness of the inner lines. We used Necker cube images with eight different values of the control parameter (Figure 1A). Half of them, *a* = {0.15, 0.25, 0.4, 0.45} were considered left-oriented and another half, *a* = {0.55, 0.6, 0.75, 0.85} were right-oriented. For *a* ≈ 0 and *a* ≈ 1, the stimulus had a clearly identified left and right orientation. For *a* ≈ 0.5, the stimulus became ambiguous. We did not use the Necker cube image with *a* = 0.5. We supposed that its processing was determined by endogenous factors rather than the stimulus features (Engel and Fries, 2010). Each Necker cube image was drawn in black and gray lines located at the center of the computer screen on a white background. A red dot at the center of the Necker cube attracted the subject's attention and prevented possible perception shifts due to eye movements while observing the image. The 14.2-cm Necker cubes were demonstrated on a 24″ BenQ LCD monitor with a spatial resolution of 1,920 × 1,080 pixels and a 60-Hz refresh rate. The subjects were located at a 70–80 cm distance from the monitor with a visual angle of ~0.25 rad.

### 2.3. Experimental Protocol

The whole experiment lasted 40 min for each participant, including 150 s recordings of the resting-state EEG before and after the main part of the session. During the main part, we presented 400 Necker cube images with predefined values for the degree of ambiguity, *a*, in random order. Each stimulus with a particular ambiguity was presented 50 times. The *i*-th stimulus was presented during a time interval τ_*i*_, followed by abstract image presentation for a time interval of γ_*i*_ (Figure 1B). We instructed the participants to identify stimulus orientation as accurately as possible. Subjects reported their decisions by pressing either the left (for a left orientation) or the right (for a right orientation) key with their left or right hand, respectively. The duration of the stimulus exhibition varied in the range of 1–1.5 s. We also applied random variation in the stimulus ambiguity. Lastly, to draw away the observer's attention and make the perception of the next stimulus independent of the previous one, different abstract pictures were exhibited for 3–5 s between subsequent demonstrations of the Necker cube images. Examples of the abstract pictures are shown in Figure 1B. For each stimulus, we estimated the behavioral response by measuring the response time (RT) as the time interval between the stimulus onset and key pressing. We also monitored the accuracy by comparing the actual stimulus orientation with the subject's response.

### 2.4. EEG Acquisition

The EEG signals were recorded using the monopolar registration method and the 10–10 electrode scheme. We recorded 31 signals with two reference electrodes, A1 and A2, on the earlobes and a ground electrode N just above the forehead. The signals were acquired via cup adhesive Ag/AgCl electrodes placed on the “Tien–20” paste (Weaver and Company, Colorado, USA). Immediately before the experiments started, we performed all necessary procedures to increase the conductivity of the participant's skin and reduce its resistance using the abrasive NuPrep gel (Weaver and Company, Colorado, USA). After the electrodes had been installed, the impedance was monitored throughout the experiments. Usually, the impedance values varied within a 2–5 kΩ interval. An Encephalan-EEG-19/26 electroencephalograph (Medicom MTD company, Taganrog, Russian Federation) with multiple EEG channels and a two-button input device (keypad) was used for amplification and analog-to-digital conversion of the EEG signals. This device had a registration certificate from the Federal Service for Supervision in Health Care, No. FCP 2007/00124, dated 07.11.2014, and European Certificate CE 538571 of the British Standards Institute (BSI).

### 2.5. EEG Analysis

The recorded EEG signals presented in proper physical units (millivolts, mV) were segmented into a set of 400 trials, where each trial was associated with a single presentation of the Necker cube included a 1.5 s interval before the presentation and a 0.5 s interval after button pressing. The raw EEG signals were filtered by a band-pass FIR filter with cut-off points at 1 and 100 Hz and by a 50-Hz notch filter via the embedded hardware-software data acquisition complex. Eye-blinking and heartbeat artifact removal was performed by Independent Component Analysis (ICA) using EEGLAB software (Delorme and Makeig, 2004). After the EEG preprocessing procedure, we excluded some trials due to the existence of high-amplitude artifacts and considered 320 trials out of the initial 400. The analyzed EEG data are available online (Maksimenko et al., 2020).

We calculated the wavelet power for each trial in the 4–40 Hz frequency band using the Morlet wavelet. The number of cycles *n* for each frequency *f* was defined as *n* = *f*. The wavelet analysis was performed in Matlab using the Fieldtrip toolbox (Oostenveld et al., 2011). The 0.5 s intervals on each side of the trial were reserved for the wavelet power calculation. As a result, we considered the wavelet power in three intervals including prestimulus baseline (from −1.0 to −0.5 s), the stimulus-related activity after stimulus presentation (from 0 to 0.5 s), and the stimulus-related activity (from RT−0.3 s to RT) preceding the response time. For the stimulus-related wavelet power, we calculated the event-related spectral perturbations (ERSP) via the baseline correction [stimulus-related activity–prestimulus baseline]/prestimulus baseline.

In this work, we were only interested in the effect of stimulus ambiguity. Therefore, to minimize the additional effect of stimulus orientation, including the lateralization effects associated with the motor response, we considered two conditions: low ambiguity (LA) stimuli, including the Necker cube images with *a* ∈ {0.15, 0.25, 0.75, 0.85}, and high ambiguity (HA) stimuli, including the Necker cube images with *a* ∈ {0.4, 0.45, 0.55, 0.6}. Each condition included 100 stimuli (25 per ambiguity, 50 per orientation).

### 2.6. Statistical Testing

Statistical analyses of the RT and the stimulus presentation time were performed on the subjects' median values. According to the results of normality tests, we applied either the paired-samples *t*-test or the Wilcoxon test to analyze the pairwise differences. RTs of males and females were compared via the independent-samples *t*-test. The statistical analysis was carried out in SPSS. The tests used and their parameters are mentioned in the results section.

Statistical analyses of the cortical activity in the space-time-frequency domain were performed on subject-level wavelet power, averaged across trials. Contrasts between conditions were tested for statistical significance using a permutation test in conjunction with cluster-based correction for multiple comparisons. Specifically, *t*-tests were performed to compare each pair of the (channel, frequency, time)-triplets. Elements that passed a threshold value corresponding to a *p*-value of 0.01 (two-tailed) were marked together with their neighboring elements and were collected into separate negative and positive clusters. The minimal number of required neighbors was set to 2. The *t*-values within each cluster were summed and rectified. These values were fed into the permutation framework as the test statistic. A cluster was considered significant when its *p*-value was below 0.025, corresponding to a false alarm rate of 0.01 in a two-tailed test. The number of permutations was 2,000. Analysis was performed in the Fieldtrip toolbox for Matlab.

## 3. Results

The subjects responded faster to LA stimuli (M = 0.86 s, SD = 0.24) than to HA stimuli (M = 1.09 s, SD = 0.3): *t*_(19)_ = 5.83, *p* < 0.001 (Figure 1C). The distribution of the pairwise differences showed that one subject demonstrated an effect in the opposite direction, responding faster to HA stimuli. The stimuli were presented randomly and, therefore, the median presentation time of HA and LA Necker cubes did not differ: *t*_(19)_ = −0.992, *p* < 0.334 (Figure 1D). The repeated-measures ANOVA used to compare RT for the similar and opposite orientation of the previous stimulus revealed an insignificant effect of the previous stimulus orientation [*F*_(1, 19)_ = 1.86, *p* = 0.188] and an insignificant interaction effect of ambiguity×orientation [*F*_(1, 19)_ = 0.434, *p* = 0.518]. Finally, there was no correlation between age and RT to HA stimuli [*r*_(20)_ = −0.24, *p* = 0.3] or LA stimuli [*r*_(20)_ = −0.31, *p* = 0.17]. RT was similar for males and females for both HA stimuli [*t*_(18)_ = 0.79, *p* = 0.436] and LA stimuli [*t*_(18)_ = 0.96, *p* = 0.348]. ER was higher for HA stimuli (M = 8.95%, SD = 11.5) than for LA stimuli (M = 1.65%, SD = 2.6): *Z* = 3.5, *p* < 0.001 via Wilcoxon test (Figure 1E). Three of the 20 subjects demonstrated no effect, and one subject demonstrated an effect in the opposite direction.

Testing the prestimulus wavelet power in the frequency range 4 − 40 Hz, the paired-samples *t*-test with cluster-based correction for multiple comparisons revealed no significant difference between HA and LA stimuli. We also compared the wavelet power between males and females and between two age-groups (“< 26 y.o.” vs. “> 26 y.o.”, where 26 was the median age) via the independent-samples *t*-test with cluster-based correction for multiple comparisons. In both cases, the difference was insignificant.

**Figure 2:**
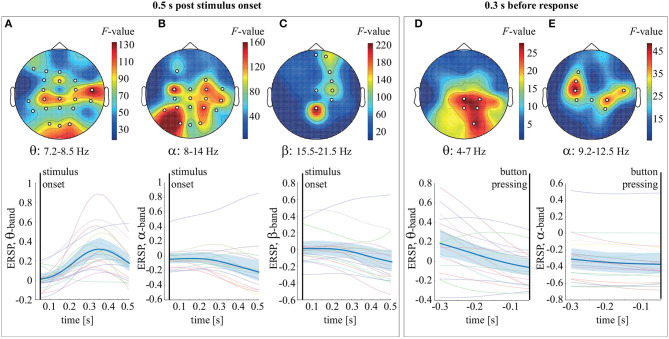
Top row: the *F*-value and the channel clusters reflecting significant change in ERSP in the 0.5 s post-stimulus onset in the θ **(A)**, α **(B)**, and β **(C)** bands and 0.3 s before response in the θ **(D)** and α **(E)** bands. Bottom row: ERSP (group mean and 95% confidence interval) averaged over the θ **(A)**, α **(B)**, and β **(C)** bands in the 0.5 s post-stimulus onset, as well as over the θ **(D)** and α **(E)** bands in the 0.3 s before response.

To analyze ERSP evolution during stimulus processing, we combined trials corresponding to LA and HA stimuli. The stimulus processing period was segmented into 0.05 s intervals. We used the dependent-samples *F*-test to compare ERSP over these intervals. Multiple comparisons correction was implemented via the cluster-based permutation test. In the 0.5 s post-stimulus onset, we observed two significant clusters with *p* = 0.00049 in the frequency bands 4–14 and 15.5–21.25 Hz. Based on these results, we defined the frequency bands of interest as 4–8 Hz (θ-band), 8–14 Hz (α-band), and 15.5–21.25 Hz (β_1_-band). For the θ-band, the observed cluster included EEG sensors in the occipital (O1, O2, Oz), parietal (P3, P4, Pz), bilateral temporal (T5, TP7, TP8, T4), parieto-central (CP3, CP4, CPz), central (C3, C4), fronto-central (FC3, FC4, FCz), and frontal (F7, F3, Fz) areas (Figure 2A). For the α-band, the cluster included the occipital (O1, O2, Oz), parietal (P3, P4, Pz), temporal (T5, T6, TP8, T4), parieto-central (CP3, CP4, CPz), central (C3, C4, Cz), right fronto-central (FC4), and left frontal (F3) sensors (Figure 2B). The β-band cluster included sensors in the parietal (Pz), right parieto-central (CP4), left-lateralized central (CP4, Cz), left fronto-central (FC4), and left frontal (F4, Fp2, Fpz) areas (Figure 2C). Analysis of ERSP averaged over these sensors and the frequency bands of interest revealed that θ-band power increased, peaking at 0.35 s post-stimulus onset (Figure 2A). The ERSP in the α and β-bands decreased gradually over the time interval considered (Figures 2B,C). In the 0.3-s interval preceding behavioral response, an *F*-test revealed two significant clusters with *p* = 0.014 and *p* = 0.001 in the θ (4–7 Hz) and α (9.2–12.5 Hz) frequency bands. The observed θ cluster included EEG sensors in the occipital and parietal areas (Figure 2D). The α cluster included sensors bilaterally in the sensorimotor area (Figure 2E). Finally, ERSP, averaged over the frequency bands and the corresponding sensors, decreased monotonically within the time-interval considered (Figures 2D,E).

**Figure 3:**
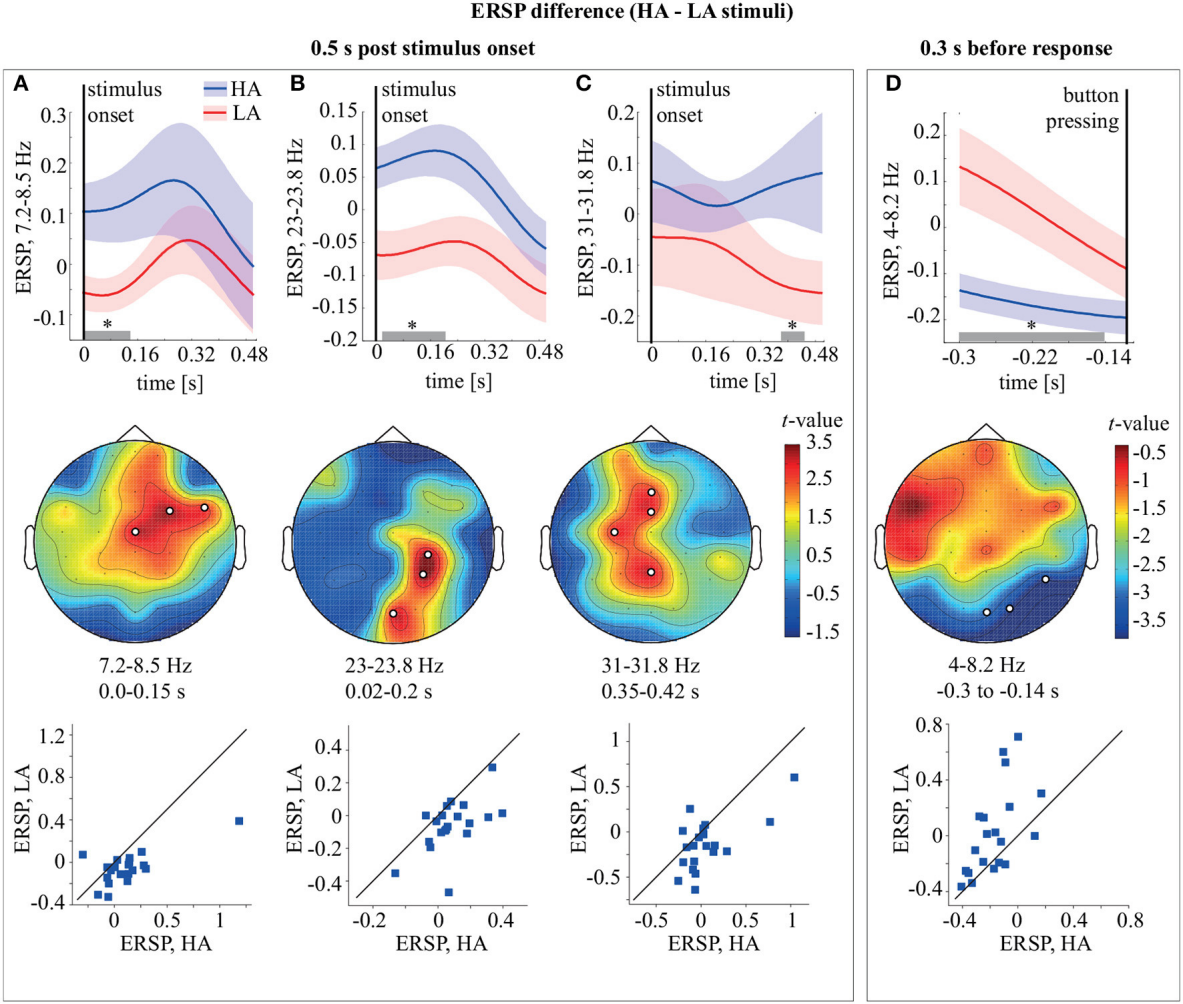
Top row: ERSP of the frontal θ-cluster **(A)**, occipito-parietal β_1_-cluster **(B)**, fronto-parietal β_2_ cluster **(C)**, and occipital θ-cluster **(D)** during HA and LA stimulus processing. Middle row: the *t*-value and the channel clusters as a result of the ERSP comparison between HA and LA stimuli in the 0.5 s post-stimulus onset and 0.3 s before the response. The subplots show the significant clusters observed in the θ **(A)**, β_1_ **(B)**, and β_2_ **(C)** frequency bands in the 0.5 s post-stimulus and in the θ **(D)** band in the 0.3 s before the response. The legends display time-frequency region for each cluster. Bottom row: scatter-plots show the ERSP difference between LA and HA stimuli for all participants. *p < 0.05 based on the paired samples *t*-test with the multiple comparison correction via the randomization technique.

Comparing ERSP during HA and LA stimulus processing for 0.5 s post-stimulus onset, we observed three significant (*p* < 0.01) positive clusters (Figure 3). The first cluster (*p* = 0.0089) extended from the stimulus onset to 0.15 s in the upper θ-frequency band 7.25–8.5 Hz and included midline central (Cz), right fronto-central (FC), and right fronto-temporal (FT) sensors (Figure 3A). The ERSP in this cluster is higher for HA stimuli in 18/20 subjects. The second cluster (*p* = 0.0049) extended from ~0.02 to 0.2 s in the β_1_-frequency band 23–23.8 Hz and included the midline occipital (O2), right parietal (P4), and parieto-central (CP4) sensors (Figure 3B). According to the distribution of pairwise differences, this cluster had higher ERSP for HA stimuli in 17/20 subjects. The third cluster (*p* = 0.0074) extended from ~0.35 to 0.42 s in the β_2_-frequency band 31–31.8 Hz and included the midline parietal (Pz), left central (C3), midline frontal (Fz), and fronto-central (FCz) sensors (Figure 3C). Sixteen of the 20 subjects demonstrated higher ERSP for HA stimuli in this cluster. Comparing ERSP between HA and LA stimuli in the 0.3 s before the response, we observed one significant cluster with *p* = 0.0084 in the 4–8.2 Hz θ-frequency band. This cluster extended for 0.3–0.114 s before the behavioral response and included occipital (Oz, O2) and right temporal (T6) EEG sensors (Figure 3D). ERSP in this cluster was higher for LA stimuli in 15/20 subjects.

## 4. Discussion

Many studies have used ambiguous visual stimuli to analyze spontaneous perceptual reversals. These involve the presentation of an ambiguous stimulus whose interpretation spontaneously alternates under endogenous or exogenous factors (Kornmeier and Bach, 2006; Yokota et al., 2014). Our paradigm excluded the presentation of completely ambiguous stimuli. Therefore, each presented stimulus had a particular interpretation that was defined by its morphology. The subjects were able to report a correct stimulus interpretation above chance, but their response time increased with increased ambiguity.

We observed that during stimulus processing, θ-band power grew over the majority of the recording electrodes, peaking at 0.35 s post-stimulus onset (Figure 2A). There is ample evidence that θ-band activity characterizes the brain's ability to transfer and coordinate information over large distances (da Silva, 2013) and prolonged periods (Kayser et al., 2012). High θ-band power confirms the critical role of large-scale networks in visual processing, providing evidence that perception depends not just on the external stimulus. Instead, the brain integrates sensory evidence with other internal constraints, including expectations, recent memories, etc. (Von Stein and Sarnthein, 2000). Thus, we supposed that increasing stimulus-related θ-band power across large-scale cortical regions coordinated information in the brain networks, including visual sensory as well as higher-order areas (Mathes et al., 2014).

The stimulus-related α-band power decreased over the EEG sensors in the parieto-occipital and sensorimotor areas (Figure 2B). Reduced stimulus-related α-band power in the occipital (visual) and parietal (attentional) areas may reflect primary visual processing and also cognitive processing and visual attention (Pfurtscheller et al., 1994). In the motor area, the most significant change of the α-band power was for the C3 and C4 electrodes, manifesting the motor preparation process. The β-band power started decreasing from 0.25 s post-stimulus onset in the fronto-parietal and sensorimotor areas (Figure 2C). There is a view that high β-band power reflects the involvement of a strong endogenous, top-down component (Engel and Fries, 2010). In particular, parietal β-band power grows during the processing of ambiguous stimuli where the percept solely relies on endogenous factors rather than stimulus features (Okazaki et al., 2008). The fronto-parietal β-band activity during the stimulus processing is a marker of top-down attentional mechanisms that control the accumulation of the decision-relevant sensory information (Buschman and Miller, 2007). We supposed that these top-down mechanisms guide the subject's attention to particular properties of the Necker cube (e.g., the contrast of the inner edges), supporting a correct decision about its orientation. The fact that β-band activity decreased after 0.25 s might evidence that the information accumulation process was complete and the perceptual ambiguity was unresolved. Finally, reduced sensorimotor β-band power usually reflects movement preparation in decision-making tasks where the choices are to be communicated via a motor response (see Spitzer and Haegens, 2017 for a literature review).

Considering the time interval preceding the behavioral response, we observed continuously reducing power in the θ and α-bands. The θ-band activity waned in the right-lateralized parietal and occipital areas until the subject had pressed the button (Figure 2D). This might show that occipito-parietal areas remained activated over the entire processing period, unlike the frontal areas, whose activity peaked during the earlier processing stage and rapidly diminished. The α-band power decreased bilaterally over the sensorimotor electrodes (Figure 2E). Thus, we supposed that in this interval, the α-band activity supported only the motor execution.

When comparing the processing of HA and LA stimuli, we observed that α-band power changed similarly regardless of the degree of stimulus ambiguity. Increasing ambiguity induced higher θ-band power over the anterior electrodes for 0.15 s post-stimulus onset (Figure 3A). Previous studies reported that anterior θ-band activity might control and influence posterior brain sites, including early visual areas, in a task requiring reliable top-down control (de Borst et al., 2012; Lee and D'Esposito, 2012; Cohen and Van Gaal, 2013). In particular, Mathes et al. (2014) showed that anterior θ-band response exceeded posterior response during an ambiguous task. The authors related the anterior maximum of the θ-band power with the prevalence of expectations and prior experience in ensuring coherent object perception when the sensory information is inconclusive and elicits an ongoing conflict between perceptual interpretations. In line with Mathes et al. (2014), we concluded that on the earlier processing stage, ambiguous stimulus processing mostly relied on top-down processes, in contrast to the processing of an unambiguous stimulus. These top-down processes might be related explicitly to expectations, memory, and perceptual conflict resolution.

Increasing stimulus ambiguity also caused higher β-band power during two different time intervals over the different brain areas. First, β-band power grew in the right occipito-parietal area for 0.02–0.2 s post-stimulus onset (Figure 3B). A previous study of ambiguous Necker cube perception by Yokota et al. (2014) revealed that the right-occipital β-band power increased for 0.1–0.15 s after the onset of a completely ambiguous stimulus only when its perception differed from that of a previous unambiguous stimulus. These results linked activity in the right occipital beta band with endogenous switching between rivaling percepts. The authors also related their findings by the visual feedback circuits affecting early visual processing within 0.1 s of stimulus onset (Foxe and Simpson, 2002). They concluded that the enhancement of early β-band activity might reflect the interaction between the visual cortex and other occipital and parietal cortical regions necessary for stimulus disambiguation. Finally, they proposed that the disambiguation process was complete within the first 0.25 s after stimulus onset. In line with Yokota et al. (2014), we supposed that high right occipito-parietal β-band power at the earlier processing stage reflected the disambiguation process.

Processing of ambiguous stimuli also resulted in higher β-band power over the parietal and midline frontal areas for 0.35–0.42 s post-stimulus onset (Figure 3C). Yokota et al. (2014) also reported increased β-band power for 0.35–0.45 s during the processing of an ambiguous stimulus. According to Pitts and Britz (2011), this late component might reflect the conscious processing of the perceptual information or maintenance of the percept in working memory. The other studies provided evidence that the demands of working memory could alter the β-band activity in the fronto-parietal cortical areas (see Dotson et al., 2014 for a literature review). However, overall changes in oscillatory activity during working memory processing are also often found in frequency bands other than β, especially θ (see Roux and Uhlhaas, 2014 for a literature review). We did not simultaneously observe higher θ-band power for the ambiguous stimuli. Therefore, we did not report an enhancement of working memory demands in the later stages of ambiguous stimulus processing.

In turn, we supposed that high fronto-parietal β-band power might reflect the decision-making process. A traditional view is that β-band activity in decision-making reflects motor preparation only, where the motor plan expresses the final step after the higher-order areas have reached a decision based on sensory input. However, several studies pointed to a more direct involvement of β-band activity in decision formation, regardless of a specific motor plan. Also, decision-related predictions in the β-frequency band can occur beyond sensorimotor regions, both within and between distributed cortical areas, including fronto-parietal circuits (see Spitzer and Haegens, 2017 for a literature review). In their recent work (Chand and Dhamala, 2017), Chand and Dhamala analyzed neural interaction between the anterior cingulate-insula network and the fronto-parietal network during decision-making tasks. They reported that the fronto-parietal network achieved control over the cingulate-insula network in the β-band during a 0.22–0.42 s timeframe in behaviorally harder decision-making tasks.

Finally, our study has potential limitations. The number of participants is small; therefore, there is a risk that the individual characteristics of the people (such as sex, age, and psychological traits) will have influenced their perception of ambiguous stimuli and decision-making (see Scocchia et al., 2014 for a literature review). For our group, we observed no gender and age effects on the reaction time and the spectral power due to the almost uniform distribution of these factors. At the same time, we expect that another group of younger or older subjects may demonstrate different scores for both behavioral and brain activity levels. The subjects' personality traits may also affect cognitive processes and behavioral performance during cognitive tasks. In particular, anxiety level is essential for the perception of ambiguous situations. Previous studies documented that people with anxiety tended to interpret ambiguous stimuli negatively (see Park et al., 2016 for the review). We assumed that the processing of an emotionally neutral Necker cube was relatively unaffected by anxiety. Furthermore, the images presented were not wholly ambiguous; therefore, their interpretation relied not entirely on endogenous factors but on the processing of the stimulus morphology. The existence of objectively decision-relevant features in the sensory information also reduces the influence of endogenous components, such as the state of the observer. Nevertheless, to ensure that the observed effects are not affected by personality traits, further studies should include a personality traits assessment beforehand. Finally, a small number of EEG channels may not be sufficient for reliable localization of the reported neuronal activity in space. Therefore, we defined spatial locations on the EEG sensor-level. Further studies should use MEG and EEG signals with a high spatial resolution to promote precise localization of the observed activity on both sensor and source levels.

## Data Availability Statement

The datasets presented in this study can be found at dx.doi.org/10.6084/m9.figshare.12292637.v2.

## Ethics Statement

The studies involving human participants were reviewed and approved by The local Research Ethics Committee of the Innopolis University. The patients/participants provided their written informed consent to participate in this study.

## Author Contributions

VM, AH, and AP conceived the study. AK and NF analyzed the behavioral and EEG data. MK performed the experiments. VM interpreted the results. VM, NF, AH, and AP wrote the manuscript. All authors contributed to the article and approved the submitted version.

## Conflict of Interest

The authors declare that the research was conducted in the absence of any commercial or financial relationships that could be construed as a potential conflict of interest.
